# Clinical Feature, Treatment, and KCNH5 Mutations in Epilepsy

**DOI:** 10.3389/fped.2022.858008

**Published:** 2022-07-07

**Authors:** Xiufu Hu, Junli Yang, Man Zhang, Tie Fang, Qin Gao, Xinjie Liu

**Affiliations:** ^1^Department of Pediatrics, Qilu Hospital of Shandong University, Jinan, China; ^2^The First Affiliated Hospital of Zhengzhou University, Zhengzhou, China; ^3^Beijing Children’s Hospital, Beijing, China; ^4^Beijing MyGenostics Co., Ltd, Beijing, China

**Keywords:** KCNH5, epilepsy, clinical manifestations, molecular characterization, treatment

## Abstract

The voltage-gated Kv10.2 potassium channel, encoded by KCNH5, is broadly expressed in mammalian tissues, including the brain. Its potential mechanism remains unclear. According to previous studies, dysfunction of Kv10.2 may be associated with epileptic encephalopathies and autism spectrum disorder (ASD). To date, only one disease-causing mutation of KCNH5 has been reported, and it involves a case that presented with seizures and autism symptoms. In this study, we discovered and characterized three *de novo* mutations in KCNH5 that potentially caused severe conditions observed in three Chinese children. All of them experienced seizures, two of them presented with epileptic encephalopathy, one of them presented with ASD, and one did not relapse after drug withdrawal. Notably, treatment with antiepileptic drugs (AEDs) was effective in all patients whose epileptic seizures were controlled. The structures of the proteins resulting from the mutations were predicted in two of the three cases. This provides powerful insight into clinical heterogeneity and genotype*-*phenotype correlation in KCNH5-related diseases.

## Introduction

Epilepsy is defined as having two or more unprovoked seizures that are characterized by recurrent episodes of paroxysmal brain dysfunction due to sudden, disorderly, and excessive neuronal discharge. According to a previous estimate, 30% of individuals with epilepsy carry pathogenic mutations (1). Most genetic epilepsies are associated with mutations in genes encoding subunits of voltage-gated ion channels, including the sodium, calcium, magnesium, and potassium channels. Nearly 80% of children with epilepsy have at least one comorbid disorder, and 43% have additional developmental/psychiatric disorders, including intellectual disability, attention deficit disorder with hyperactivity (ADHD), behavioral/emotional disorders, and autism (2). Autism spectrum disorder (ASD) is a neurodevelopmental disorder characterized by social impairments and communication difficulties. At present, ASD etiology is not well understood; however, genetic factors play an important role. Approximately 40% of ASD cases can be attributed definitively to genetic mutations with more than 800 ASD predisposition genes identified to date (3, 4). *Via* the whole-genome sequencing (WGS) of 208 genomes from 53 families affected by simplex autism, researchers suggest that smaller, often multiple [Copy number variations (CNVs)] and gene mutations are important factors in simple ASD (5). Comorbidities are very common in ASD, and approximately 8–30% of patients with ASD suffer from epilepsy, which is frequently caused by mutations in susceptibility genes, such as MECP2, FMR1, TSC, PTEN, and SHANK3 (4, 6–11). According to previous studies, the dysfunction of Kv10.2 may be associated with epileptic encephalopathies and ASD. We report three *de novo* mutations in KCNH5, which encodes the voltage-gated Kv10.2 potassium channel in three Chinese children (Table 1). All of them experienced seizures, two of them presented with developmental disorders, one of them experienced ASD, and one did not relapse after drug withdrawal. The structures of the proteins resulting from the mutations were predicted in two of the three cases. To the best of our knowledge, a variant of KCNH5 has not been reported since the first case in 2013 (1).

**TABLE 1:** Clinical features of reported individuals with KCNH5 pathogenic variants.

Patient	Sex	Age at the time of reporting	Age at seizure onset	Pathogenic variant	Initial seizure type(s)	EEG findings and evolution	Craniocerebral MRI	Comorbid disorder	AEDs
The first report	Male	13 years	6 months	c.980G > A (p.R327H)	Non-febrile GTCS	Frequent multifocal spikes, almost continuous during sleep, even with good seizure control	N/A	ASD; epileptic encephalopathy; language delay	Valproic acid
Case 1	Male	4 years	8 months	c.980G > A (p.R327H)	Non-febrile GTCS	Wide high 2–3 Hz periodic sharp wave complexes	Normal	ASD; language delay; bad behavior control	Valproic acid and lamotrigine
Case 2	Female	4 years	6 months	c.2020-4A > G (splicing)	Non-febrile eclampsia nutation	High-amplitude slow waves and spikes or sharp waves occurring irregularly	Normal	Psychomotor development delay	Vigabatrin and valproic acid
Case 3	Male	2 years	7 months	c.962G > A (p.S321N)	Non-febrile clonic seizures	Normal	Normal	None	Valproic acid

## Materials and Methods

After obtaining informed consent from the patients’ parents, the clinical features and date were examined, and genetic molecular analysis was performed.

### DNA Library Preparation

A DNA Sample Prep Reagent Set (Beijing MyGenostics Co., Ltd., Beijing, China) was used for the preparation of standard Illumina libraries, including end repair, adapter ligation, and PCR amplification, which were further sequenced by DNBSEQ (DNBSEQ-T7).

### Enrichment and Sequencing of Targeted Genes

The amplified DNA was captured using a GenCap whole-exome sequencing (WES) kit (MyGenostics Inc., Beijing, China). The biotinylated 100 bp capture probes were designed to tile, along with the coding exons and 50 bp flanking regions of all the genes. The capture experiment was conducted according to the manufacturer’s protocol. Briefly, a DNA library of 500 ng was first mixed with Buffer BL and GenCap gene panel probes (MyGenostics Inc., Beijing, China). The mixture was heated at 95°C for 5 min, and then, at 65°C for 5 min, on a PCR machine. After that, 19 μl of 65°C pre-warmed Buffer HY (MyGenostics, Baltimore, MD, United States) was added to the mixture, and this mixture was held at 65°C with the PCR lid heating for 16–24 h for hybridization. Fifty microliter of MyOne beads (Life Technology) were washed using 50 μl of 1X binding buffer three times, and then, resuspended in 50 μl of 1X binding buffer. Then, the hybrid mixture was washed with WB1 buffer at room temperature for 15 min once and WB3 buffer at 65°C for 10 min three times. The bound DNA was eluted with a buffer and amplified for 13 cycles using the following program: 95°C for 4 min (1 cycle); 98°C for 30 s, 65°C for 30 s, and 72°C for 30 s (13 cycles); and 72°C for 5 min (1 cycle). The PCR product was purified using SPRI beads (Beckman Coulter) according to the manufacturer’s protocol. The enrichment libraries were sequenced on a DNBSEQ-T7 sequencer for a paired reading of 150 bp.

### Bioinformatics Analysis

After sequencing, the raw data were saved in FASTQ format. Both Illumina sequencing adapters and low-quality reads (<80 bp) were filtered by cutadapt software^1^. The clean reads were mapped to the UCSC hg19 human reference genome using the parameter BWA of Sentieon software^2^. The duplicated reads were removed using the parameter driver of Sentieon software, and the parameter driver was used to correct the base. This was done so that the quality of the base in the reads of the final BAM file could be closer to the real probability of mismatch with the reference genome, and the mapped reads were used for the detection of variation. The variants of SNP and InDel were detected by the parameter driver of Sentieon software. Then, the data were converted to VCF format. Variants were further annotated by ANNOVAR software^3^, associated with multiple databases [e.g., 1,000 Genomes, ESP6500, dbSNP, ExAC, In-house (MyGenostics), and HGMD], and predicted by SIFT, PolyPhen-2, MutationTaster, and GERP++.

### Variant Selection

In this study, four steps were used to select the potential pathogenic mutations in downstream analysis: (i) mutation reads should be greater than 5, and the mutation ratio should be no less than 30%; (ii) the mutations should be removed when the frequency of mutation is more than 5% in the 1,000 Genomes, ESP6500, and In-house databases; (iii) the mutations should be dropped if they are in the InNormal database (MyGenostics); and (iv) the synonymous mutations should be removed if they are not in the HGMD database. Afterward, any remaining mutations should be potential pathogenic mutations for further analysis.

The SWISS-MODEL workspace^4^ was used to characterize the effect of the mutations on the protein. Conservation of the KCNH5 gene in different species was analyzed by UGENE software^5^.

## Results

### Case 1

**FIGURE 1:**
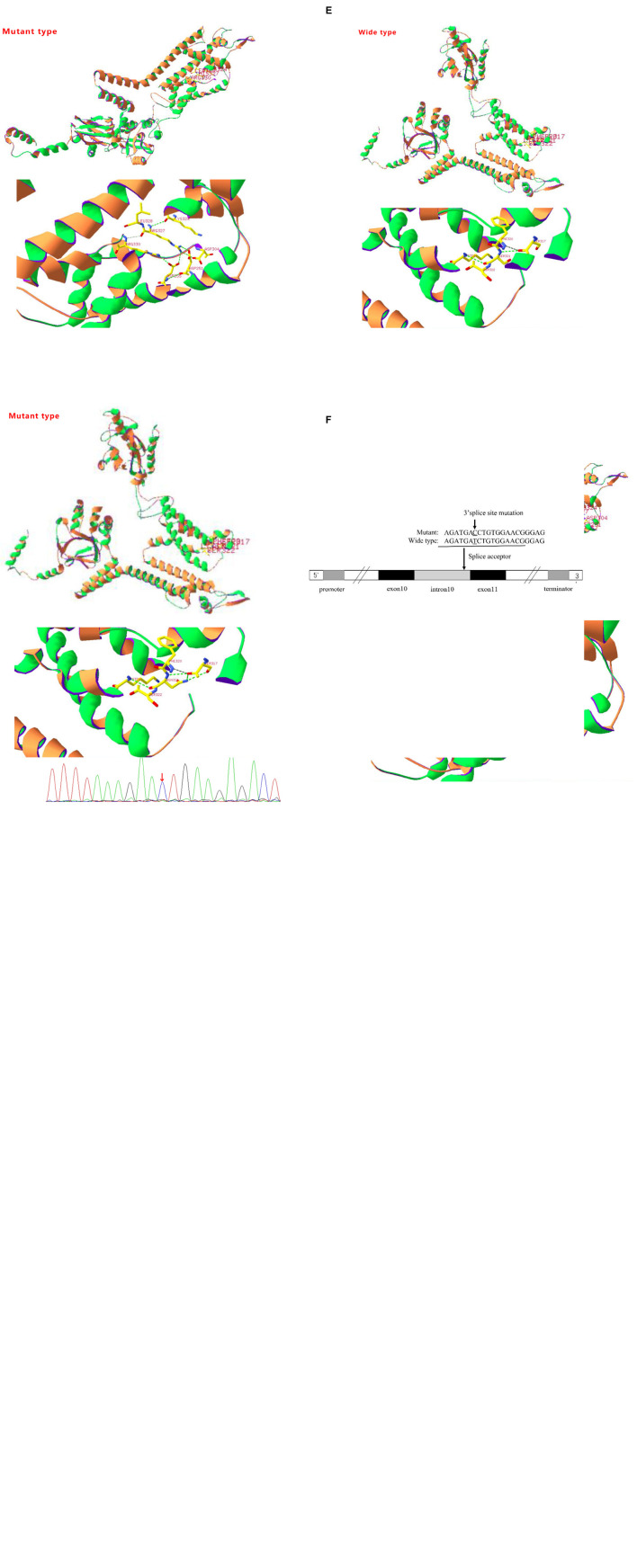
**(A)** Sanger sequencing results of the KCNH5 gene in the case 1 family. One mutation of KCNH5 was identified in this patient, which was c.980G > A (Red arrow). The parents are normal. **(B)** Sanger sequencing results of the KCNH5 gene in the case 2 family. One mutation of KCNH5 was identified in this patient, which was c.2020-4A > G (Red arrow). The parents are normal. **(C)** Sanger sequencing results of the KCNH5 gene in the case 3 family. One mutation of KCNH5 was identified in this patient, which was c.962G > A (Red arrow). The parents are normal. **(D,E)** Mutation analysis and models of the predicted protein structure of KCNH5. **(A–C)** Show the mutations in the patients and their parents, as confirmed by Sanger sequencing. **(D)** Shows the 3D structure of KCNH5 in case one (c.980G > A). The green dotted line indicates hydrogen bonds between groups. The solid yellow line represents the covalent bonds. In the wild type, the main body of Arg327 is connected to LYS324 and Arg330 by hydrogen bonds, and the side chain of Arg327 is connected to Asp304, Asp251, and Asp255 by hydrogen bonds. In the mutant type, the main body of His327 is connected to LYS324 and Arg330 by hydrogen bonds, and the side chain of His327 is connected to Arg330 by hydrogen bonds. **(E)** Shows the 3D structure of KCNH5 in case 3 (c.962G > A). In the wild type, the main body of Ser321 is connected to LYS324 and Ser317 by hydrogen bonds. In the mutant type, the main body of Asn321 is connected to LYS324 and Ser317 by hydrogen bonds, and the side chain of Asn321 is connected to Ser317 by hydrogen bonds. **(F)** The mutation are of case 2.

In the first case, a G1P1 boy was born to a non-consanguineous family at 39 weeks with a birth weight of 3.7 kg. The boy first experienced seizures at the age of 8 months. He presented with tonic-clonic seizures without a fever. Electroencephalography (EEG) showed wide, high, and sharp 2–3 Hz periodic wave complexes. Meanwhile, he also had trouble being in contact with people. The autism behavior checklist indicated that the child had autism-like symptoms (69 points). Craniocerebral MRI was normal. He was treated with antiepileptic drugs (AEDs) after 3 or 4 seizures. The initial treatment included clonazepam (0.1 mg/kg/d) and valproic acid (30 mg/kg/d). He was seizure-free for 2 years until his parents stopped administering his medication. The child suffered from tonic-clonic seizures again when he was 3 years old. Compared with that of children of the same age, his ability to express himself was poor. Thereafter, genetic testing was finally performed. A *de novo* variant c.980G > A (p.R327H) of KCNH5 was identified in the patient *via* a gene mutation analysis of the boy and his parents by using targeted whole-exome next-generation sequencing (Figure 1A), which was rated as pathogenic according to the American College of Medical Genetics and Genomics. The SWISS-MODEL workspace (see text footnote 4) was used to characterize the effect of the mutations on the KCNH5 protein. The structure of the protein after the mutation was predicted and compared with that of the wild type (Figure 1D). The patient was medicated with valproic acid and lamotrigine to treat the seizures that occurred as a result of stopping the antiepileptics. The child is 4 years old now and has been seizure-free for nearly 2 years with the treatment of valproic acid (30 mg/kg/d) and lamotrigine (2 mg/kg/d). Additionally, his autism syndrome is partially controlled via rehabilitation training.

### Case 2

The next case, G1P1, is that of a girl who was born to a non-consanguineous family at 38 + 4 weeks with a birth weight of 3.5 kg. The girl first experienced seizures at the age of 6 months. She presented with non-febrile eclampsia nutation. The girl was able to raise her hand, but she would sit at that time. Her seizure frequency ranged from 1 time every 4–5 days to more than once per day overtime. At the age of 8 months, the girl was sent to the hospital and underwent systematic testing. Cranial MRI showed no abnormality, and EEG indicated high-amplitude, slow waves, and spikes or sharp waves occurring irregularly. She was diagnosed with infantile spasm syndrome and received vigabatrin and valproic acid therapy (20 mg/kg/d). She had good seizure control for 2 years until she stopped taking valproic acid. Then, she presented with a partial seizure. The EEG pattern was indicative of West syndrome. The patient restarted the combination of valproate acid (30 mg/kg/d) and vigabatrin (100 mg/kg/d) and received ACTH therapy (25 IU/d*4 W). She is now 4 years old and has been seizure-free for 2 years. However, she still demonstrates developmental delay, especially in her language skills. The WES found a *de novo* variant c.2020-4A > G (splicing) of KCNH5 (Figure 1B). The variant indicates that the fourth base from last, involved in coding the intron (intron 10) between the 2019 and 2020 nucleotides, mutated from C to T (Figure 1F). The mutation occurs at the splice receptor site of the exon 11. It was rated to be likely pathogenic according to the American College of Medical Genetics and Genomics. The mutation c.2020-4A > G was predicted to be disease-causing by MutationTaster software. The mutation might affect protein features and change splice sites^6^.

### Case 3

Case 3 is that of a 4-year-old, G1P1 boy. He was born to a non-consanguineous family at 40 + 2 weeks with a birth weight of 3.85 kg. The boy first experienced seizures at the age of 7 months. He presented with non-febrile clonic seizures without comorbidities. However, EEG and craniocerebral MRI revealed no abnormalities. After 4–5 seizures occurred at 8 months, he was treated with valproate acid. The treatment was stopped after 2 months. He has now been seizure-free for approximately 3 years. A novel variant c.962G > A (p.S321N) of KCNH5 (Figure 1C) was identified in the patient *via* WES. The results of three bioinformatics prediction software programs (SIFT, PolyPhen-2, and REVEL) were benign and harmful, respectively. By verification of the pedigree, the variant was rated to be likely pathogenic. The structure of the protein resulting from the mutation was predicted (Figure 1E).

## Discussion

Ether-à-go-go (EAG) are types of voltage-gated potassium (Kv) channels. Similar to all potassium channels, their function is to bring the membrane potential closer to the potassium equilibrium potential. The voltage-gated Kv10.2 potassium channels belong to the voltage-gated potassium channels of the EAG family (12), which are expressed broadly in organs or tissues such as the brain, heart, lung, liver, and skeletal muscle (13). Previous studies have observed that Kv10.2, which is overexpressed in renal cell carcinoma, melanoma, pancreatic cancer, and medulloblastoma, can be a potential target for cancer therapy. In central nervous system diseases, the pathogenicity of a variant of KCNH5 is poorly understood. Liu et al. used a kainic acid (KA)-induced temporal lobe epilepsy model to examine the role of the Kv10.2 gene in status epilepticus (SE). The results indicated that anxiety-like behavior in rats increased after the injection of lentiviral plasmids containing the coding sequence region of the KCNH5 gene (LV-KCNH5) (14). Whole-exome sequencing (WES) is a powerful new tool for disease gene discovery. In 2013, *via* WES, Veeramah et al. reported a case of epileptic encephalopathy with seizures intractable to medical therapy and autistic features due to a KCNH5 (p.R327H) mutation. The symptoms of the case were partly controlled after valproate acid treatment (15). In a follow*-*up study, researchers found that the R327H mutation weakens ionic interactions between residue 327 and negatively charged residues, thus triggering channel opening. Voltage clamp analysis also supports the above conclusions (16). However, there are no case reports of a KCNH5 mutation after that.

**FIGURE 2:**
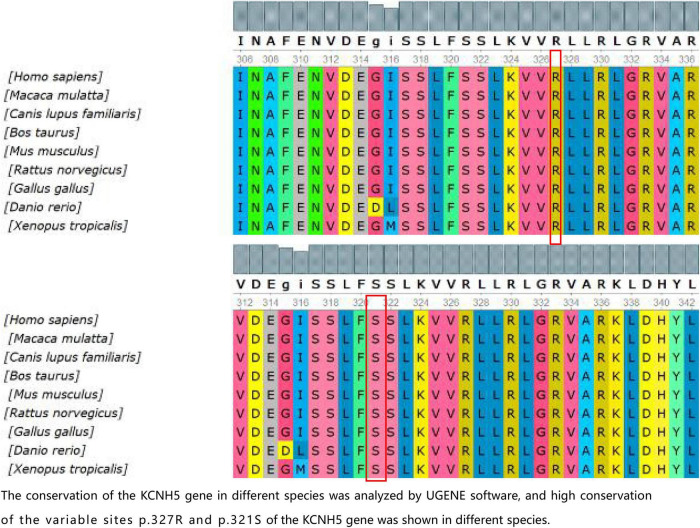
The conservation of the KCNH5 gene in different species was analyzed by UGENE software, and the high conservation of the variable sites p.327R and p.321S of the KCNH5 gene was shown in different species.

In the present study, we found three *de novo* mutations of KCNH5 in three Chinese children *via* WES, and the mutations are being reported for the first time, except for one previously published study (p.R327H). The case with the variant c.980G > A (p.R327H) of KCNH5 presented with tonic-clonic seizures with language delay and ASD. With the treatment of valproic acid and lamotrigine, the patient’s syndromes are now effectively controlled. The variant gene was rated to be pathogenic according to the American College of Medical Genetics and Genomics. The patient with the variant c.2020-4A > G (splicing) of KCNH5 was diagnosed with West syndrome. She has been seizure-free for 2 years following vigabatrin and valproic acid therapy. However, she still demonstrates developmental delay, especially in her language. It was rated to be likely pathogenic according to the American College of Medical Genetics and Genomics. The patient with the variant c.962G > A (p.S321N) of KCNH5 presented with non-febrile clonic seizures without comorbidities. He has been seizure-free after 2 months of valproic acid treatment. By verification of the pedigree, the variant was rated to be likely pathogenic. We predicted the structures of the proteins resulting from the mutations c.962G > A (p.S321N) and c.980G > A (p.R327H) of KCNH5. The conservation of the KCNH5 gene in different species was analyzed by UGENE software, which showed high conservation of the KCNH5 gene in most species (Figure 2).

In summary, the patients of KCNH5 mutation-related epilepsy presented with ASD or psychomotor development delay, except for the patient with the variant c.962G > A (p.S321N) of KCNH5 who presented with seizures without comorbidities. As previously reported, the patient with the variant c.980G > A (p.R327H) of KCNH5 presented with seizures and ASD. The seizures of the three cases were well controlled with AEDs. This provides powerful insight into the *clinical* heterogeneity and genotype-phenotype correlations in KCNH5-related diseases.

## Data Availability Statement

The datasets presented in this article are not readily available due to government restrictions. Requests to access the datasets should be directed to the corresponding author: huxiufudeyouxiang@163.com.

## Ethics Statement

The authors obtained written informed consent from the parents of the patient for this publication. Written informed consent was obtained from the minor(s) legal guardian/next of kin for the publication of any potentially identifiable images or data included in this article.

## Author Contributions

XL contributed to the conception of the work and revised it critically for important intellectual content. XH and JY acquired and analyzed the data and drafted the work, they contributed equally to this work. MZ and TF contributed to the collation of patients’ clinical data. QG provided technical and methodological support. All authors approved the publication of the content.

## Conflict of Interest

QG was employed by company Beijing MyGenostics Co., Ltd. The remaining authors declare that the research was conducted in the absence of any commercial or financial relationships that could be construed as a potential conflict of interest.

## Publisher’s Note

All claims expressed in this article are solely those of the authors and do not necessarily represent those of their affiliated organizations, or those of the publisher, the editors and the reviewers. Any product that may be evaluated in this article, or claim that may be made by its manufacturer, is not guaranteed or endorsed by the publisher.

## Footnotes

1
http://code.google.com/p/cutadapt/

2
https://www.sentieon.com/

3
http://annovar.openbioinformatics.org/en/latest/

4
http://swissmodel.expasy.org

5
http://ugene.net/

6
https://www.mutationtaster.org
